# An investigation into the acceptability, adoption, appropriateness, feasibility, and fidelity of implementation strategies for birth companionship in Tehran: a qualitative inquiry on mitigating mistreatment of women during childbirth

**DOI:** 10.1186/s12889-024-18751-z

**Published:** 2024-05-13

**Authors:** Marjan Mirzania, Elham Shakibazadeh, Sedigheh Hantoushzadeh, Zahra Panahi, Meghan A. Bohren, Abdoljavad Khajavi

**Affiliations:** 1 Department of Health Education and Promotion, School of Public Health, Tehran University of Medical Sciences, Tehran, Iran; 2 Health Information Management Research Center, Tehran University of Medical Sciences, Tehran, Iran; 3 Department of Obstetrics and Gynecology, School of Medicine, Vali-E-Asr Reproductive Health research Center, Family Health Research Institute, Tehran University of Medical Sciences, Tehran, Iran; 4 Department of Obstetrics and Gynecology, Maternal-Fetal Neonatal Research Center, Tehran University of Medical Sciences, Valiasr Hospital, Tehran, Iran; 5 Gender and Women’s Health Unit, Nossal Institute for Global Health, School of Population and Global Health, University of Melbourne, Carlton, VIC Australia; 6 Department of Social Medicine, School of Medicine, Gonabad University of Medical Sciences, Gonabad, Iran

**Keywords:** Birth companion, Implementation research, Implementation strategies, Implementation outcomes, Mistreatment, Qualitative research, Respectful maternity care

## Abstract

**Background:**

A birth companion is a powerful mechanism for preventing mistreatment during childbirth and is a key component of respectful maternity care (RMC). Despite a growing body of evidence supporting the benefits of birth companions in enhancing the quality of care and birth experience, the successful implementation of this practice continues to be a challenge, particularly in developing countries. Our aim was to investigate the acceptability, adoption, appropriateness, feasibility, and fidelity of implementation strategies for birth companions to mitigate the mistreatment of women during childbirth in Tehran.

**Methods:**

This exploratory descriptive qualitative study was conducted between April and August 2023 at Valiasr Hospital in Tehran, Iran. Fifty-two face-to-face in-depth interviews were conducted with a purposive sample of women, birth companions, and maternity healthcare providers. Interviews were audio-recorded, transcribed verbatim, and analyzed using content analysis, with a deductive approach based on the Implementation Outcomes Framework in the MAXQDA 18.

**Results:**

Participants found the implemented program to be acceptable and beneficial, however the implementation team noticed that some healthcare providers were initially reluctant to support it and perceived it as an additional burden. However, its adoption has increased over time. Healthcare providers felt that the program was appropriate and feasible, and it improved satisfaction with care and the birth experience. Participants, however, highlighted several issues that need to be addressed. These include the need for training birth companions prior to entering the maternity hospital, informing women about the role of birth companions, assigning a dedicated midwife to provide training, and addressing any physical infrastructure concerns.

**Conclusion:**

Despite some issues raised by the participants, the acceptability, adoption, appropriateness, feasibility, and fidelity of the implementation strategies for birth companions to mitigate the mistreatment of women during childbirth were well received. Future research should explore the sustainability of this program. The findings of this study can be used to support the implementation of birth companions in countries with comparable circumstances.

**Supplementary Information:**

The online version contains supplementary material available at 10.1186/s12889-024-18751-z.

## Background

Despite every woman’s right to have a positive birth experience, the mistreatment during childbirth has been documented worldwide in health facilities [1–4]. Recent studies from Iran have reported a high rate of mistreatment, including verbal abuse, frequent and painful vaginal examinations, neglect and abandonment, lack of supportive care, physical abuse [5], denial of mobility [5–7], and pain relief [5, 8]. Additionally, women are typically not allowed to choose their labour positions [6] or have a birth companion [7].

A powerful mechanism to prevent mistreatment during childbirth, as demonstrated in previous research, is the presence of a birth companion [6, 9, 10]. The World Health Organization (WHO) recommends ensuring the presence of a chosen companion during labour and childbirth, as outlined in three guidelines [11–13]. This practice is recognized as a significant strategy for enhancing the quality of care and the birthing experience [12], and is considered a crucial element of respectful maternity care (RMC) [14]. Evidence shows that having birth companions is associated with reduced pain intensity and duration of labour, increased likelihood of spontaneous vaginal birth, decreased need for analgesia, episiotomy, and cesarean section, improved birth experience, early initiation of breastfeeding, and reduced postpartum depression [15–17]. Despite recognizing these benefits, the successful implementation of birth companions remains a challenge. Many women in health facilities across the world, particularly in developing countries, are denied this right [18–23].

Addressing the research-to-practice gap and scaling up evidence-based interventions (EBIs) are key goals of implementation science (IS). IS is a multidisciplinary field defined as “the scientific study of methods to promote the systematic uptake of research findings and other evidence-based practices into routine practice, and hence, to improve the quality and effectiveness of health services” [24]. A wide range of implementation frameworks has been published. The implementation outcomes framework, introduced by Proctor et al. (2011), is one of these frameworks. This evaluation framework includes eight outcomes that serve as indicators of successful implementation: acceptability, adoption, appropriateness, feasibility, fidelity, implementation costs, penetration, and sustainability [25].

In Iran, the Ministry of Health and Medical Education (MOHME) implemented a policy in 2014 to promote maternal and newborn health by encouraging vaginal childbirth in public hospitals. One strategy of this policy to enhance the childbirth experience is the redesign of maternity wards to allow for the presence of birth companions [26]. However, public hospitals do not always support the implementation of birth companionship. As part of a large implementation research project, we have identified the challenges of implementing a birth companion as a formative research. The results showed that the major challenges include the lack of knowledge of companions, interference of companions in the clinical duties of staff, cultural issues, staff unwillingness, lack of supervision, and structural characteristics such as lack of physical space [27]. To address these issues, we developed and implemented strategies for birth companions. To the best of our knowledge, no comprehensive study has examined the implementation outcomes of birth companions in Iran. Therefore, this study aimed to investigate the acceptability, adoption, appropriateness, feasibility, and fidelity of implementation strategies for birth companions to mitigate the mistreatment of women during childbirth in Tehran.

## Methods

### Study design and setting

This study was part of a larger implementation research project examining the development and implementation of a context-specific intervention to reduce disrespectful maternity care and evaluation of strategies to improve implementation. This project, initiated in October 2021, consists of five phases: (1) needs assessment, (2) identifying the interventions to reduce mistreatment of women during childbirth, (3) identifying the implementation challenges of interventions, (4) designing implementation strategies for the intervention, and (5) testing implementation strategies in a real-life setting. The results of phases 1 and 3 of the project are presented in detail elsewhere [5, 27, 28]. This study used an exploratory descriptive qualitative design. It employed face-to-face in-depth interviews as data collection methods. Data was analyzed according to content analysis with a deductive approach.

### Study context

This study was conducted between April and August 2023 at Valiasr Hospital in Tehran, Iran. We selected this hospital because it is a major, tertiary referral hospital in Tehran that offers a wide range of obstetric services to diverse groups of women. The maternity ward, which supports approximately 200 women giving birth per month, consists of a 12-bed hall for the first stage of labour and a separate room with one bed for the active stage of labour.

### Designing implementation strategies of birth companions

In response to the challenges identified for the presence of birth companions in phase 3 of the project, we designed implementation strategies. These strategies include: (1) determining the implementation team, (2) training midwives, (3) conducting orientation sessions for obstetricians and residents, (4) training birth companions, (5) allowing birth companions to accompany women during labour and childbirth, and (6) continuously monitoring the implementation process. The implementation of these strategies spanned an 8-week period from April to June 2023. Our study focused on the acceptability, adoption, appropriateness, feasibility, and fidelity of implementation strategies for birth companions during the early implementation phase. These indicators are crucial for the initial stages of implementing health interventions [25]. According to the implementation outcomes framework of Proctor et al. (2011), acceptability is defined as “the perception among implementation stakeholders that a given treatment, service, practice, or innovation is agreeable, palatable, or satisfactory”; adoption as “the intention, initial decision, or action to employ an innovation”; appropriateness as “the degree of compatibility or perceived fit of the innovation”; feasibility as “the degree of successful implementation of the innovation in a setting”; and fidelity as “the degree of implementation of the innovation as intended” [25]. The details of implementation strategies of birth companions are provided below.

#### Determining the implementation team

The team consisted of members of the study team, the head of obstetrics, and maternity healthcare providers (MHCPs). The members of the study team (first and second authors) held a meeting with the head of obstetrics and the matron-in-charge to explain the purpose of the study.

#### Training midwives

All midwives received training from the matron-in-charge (*n* = 30, five midwives in each session). The training focused on the purpose of the study, the benefits of having birth companions during labour and childbirth, and specifically on providing training to birth companions. A member of the study team (the lead researcher) participated in the sessions.

#### Conducting orientation sessions for obstetricians and residents

The head of obstetrics held a meeting with obstetricians and residents to explain the purpose of the study and the benefits of having birth companions during labour and childbirth.

#### Training birth companions

Each birth companion received a 10-minute training session from midwives on supportive labour techniques, their roles and responsibilities during labour and childbirth, and the maternity regulations upon arrival at the maternity hospital for birth.

#### Allowing birth companions to accompany women during labour and childbirth

Any female birth companion that labouring women wanted was allowed to stay with her during labour and childbirth.

#### Continuously monitoring the implementation process

Supervisory visits to the maternity hospital were conducted by the study team, the matron-in-charge, and a team from the MOHME to oversee the implementation. The first author was present at the maternity hospital every day during both morning and evening shifts. The matron-in-charge visited the maternity hospital daily, and the third author visited the maternity hospital on a weekly basis, specifically on Fridays.

### Recruitment and participants

Three groups of participants were identified for this study: (a) women, (b) birth companions, and (c) MHCPs (midwives, residents, and head of obstetrics). The eligibility criteria were as follows: women who had a vaginal birth, regardless of the outcome; female birth companions stayed with women during labour and childbirth; residents who had completed at least one semester (six months) in the maternity hospital; and midwives and head of obstetrics with at least one year of work experience in their role and involvement in the birth companion study. Women who had a labour progress disorder and cesarean section were excluded from this study. A purposive sampling technique with maximum variation was used to recruit participants. This technique aimed to include individuals with diverse characteristics, such as age, education, socioeconomic status for women and birth companions, and age, work experience, and shift for MHCPs.

Following prior coordination and permission from the hospital authorities, the first author (M.M.) invited participants to contribute in person. The purpose and reasons for conducting the study were explained to participants. All participants provided written consent to participate in the study and audio recordings p the interviews. They were also aware that their participation was voluntary, and that they could decline or stop the interviews at any time without facing any consequences.

### Data collection

A semi-structured interview guide and face-to-face in-depth interviews were used to collect data. The interview guides were developed based on the framework of Proctor et al. [25] and then pilot-tested by conducting three initial interviews with participants, but were not analyze (Additional file 1: Interview guides). For women and birth companions, the study examined the acceptability and adoption of having a birth companion. Meanwhile the MHCPS were asked about the acceptability, adoption, appropriateness, feasibility, and fidelity of having a birth companion. Each interview started with an overarching question such as “Please describe your overall experience with the implementation of the birth companion program at this hospital”. The interview process continued with questions such as “Are you satisfied or dissatisfied with the current implementation of the program or intervention?”, “How appropriate is the implementation of this program or intervention in the hospital?”, “What are your thoughts on integrating this program or intervention into your hospital?”. Probing questions, such as ‘“Can you explain more?”, “Why do you think that is?” and ‘What would need to change?, were used. All interviews were conducted in Persian by the first author (M.M.), a PhD candidate in Health Education and Promotion with experience in conducting qualitative research. No prior relationships existed between her and any of the other participants. Interviews with the women and birth companions were conducted before discharge in a quiet and private place in the postpartum ward. Interviews with MHCPs were conducted in a private room with no one else present at the maternity hospital. The interviews lasted approximately 30–40 minutes, and field notes were taken. Each participant was contacted once during the study. At the end of each interview, demographic information of the participants was collected. Data saturation was achieved through interviews with 22 women, 14 birth companions, and 16 MHCPs, after which, no new major themes emerged.

### Data analysis

Data analysis was conducted simultaneously with data collection, using content analysis with a deductive approach [29]. First, M.M. listened to the recorded interviews repeatedly and transcribed them verbatim in Persian. Anonymity was ensured using numerical labels for each transcript file. The transcripts were checked for accuracy by the second author (E.Sh., a female professor in health education and promotion with experience in qualitative research). They were then independently coded by M.M. and E.Sh. We marked the segments of interest in the text and color-coded them. We then put these color-coded text segments together and assigned codes to them. We grouped the various codes according to their similarities and differences and linked them to pre-determined categorizations in different themes and sub-themes. The differences among coders regarding coding were discussed until a consensus was reached. Data management and analysis were performed using MAXQDA 18 software [30]. The selected quotations were translated into English to complement the findings of the study.

### Rigor

The trustworthiness of the study was assessed using Lincoln and Guba’s criteria [31]. Credibility was ensured through the triangulation of participants, including women, birth companions, and MHCPs. Additionally, the initially extracted codes were provided to three participants for approval, further enhancing credibility. Confirmability ensured by utilizing multiple data sources such as field notes, observations, audio recordings, and transcripts. Additionally, the data analysis process was reviewed and confirmed by an expert qualitative researcher who was not involved in the study. To enhance dependability, two authors independently analyzed the interviews. Furthermore, a detailed description of the research process was provided to ensure the transferability of the results. This allows for the evaluation and application of the study in different contexts. The study was reported according to the consolidated criteria for reporting qualitative research (COREQ) checklist [32] (Additional file 2: COREQ Checklist).

### Review author reflexivity

The authors maintained a reflexive stance throughout the study from study selection to data synthesis. The author team represents diverse international academic and professional backgrounds (health education and promotion, reproductive health, obstetrics and gynecology, and health services management) with a range of research focus areas and expertise. We are mindful that the authors’ perspectives might have affected the manner in which the data were collected, analyzed, and interpreted. The different perspectives of the authors could be related to their subject expertise, professional backgrounds, and knowledge of birth companionship and respectful care. As a multidisciplinary team, the authors challenged and critiqued their preconceived assumptions through reflective dialogue and supported each other to understand how these assumptions affected the analysis or interpretation of the findings. We believe that the diversity in our team helped us to critique and challenge our biases and develop the findings of the study.

## Results

### Socio-demographic characteristics of participants

A total of 52 interviews were conducted, including 22 with women, 14 with birth companions and 16 with MHCPs. The socio-demographic characteristics of the participants are summarized in Tables 1 and 2. None potential participants declined to participate in this study. Most of the women in this study were Iranians housewives with secondary education. More than one-third of the birth companions were mothers of women and most of the support was provided only during labour. We reported on the acceptability, adoption, appropriateness, feasibility, and fidelity of birth companions’ implementation strategies, using direct quotations from the participants (Table 3).

**Table 1:** Socio-demographic characteristics of women and birth companions

Characteristics	*n*	%
Women		
Number	22	100.0
Age (years)		
15–24	10	45.5
25–34	9	40.9
35–44	3	13.6
Mean ± SD = 26.36 ± 6.48		
Education		
None	1	4.5
Primary	3	13.6
Secondary	15	68.2
College	3	13.6
Employment status		
Housewife	20	90.9
Employee	2	9.1
Nationality		
Iranian	17	77.3
Afghan	5	22.7
Family income (self-report)		
Low	1	4.5
Middle	20	90.9
High	1	4.5
Gravidity		
1	11	50.0
2	4	18.2
≥ 3	7	31.8
Number of living children (including most recent birth)		
0–1	13	59.1
2–3	8	36.4
≥ 4	1	4.5
Education of husband		
Primary	1	4.5
Secondary	15	68.2
College	6	27.3
Occupation of husband		
Government employee	1	4.5
Private employee	1	4.5
Self-employed	20	90.9
Birth companions		
Number	14	100.0
Age (years)		
18–27	3	21.4
28–37	4	28.6
≥ 38	7	50.0
Mean ± SD = 38.78 ± 11.36		
Support person		
Mother	6	42.9
Sister	3	21.4
Other	5	35.7
Education		
None	4	28.6
Primary	2	14.3
Secondary	7	50.0
College	1	7.1
Employment status		
Housewife	11	78.6
Employee	3	21.4
Timing of support		
During labour only	12	85.7
During labour and childbirth	2	14.3

SD: Standard Deviation; Other: includes Husband’s mother, sister-in-law, etc

**Table 2:** Socio-demographic characteristics of maternity healthcare providers

Characteristics	*n*	%
Number	16	100.0
Age (years)		
< 30	2	12.5
30–39	8	50.0
40–49	6	37.5
Mean ± SD = 36.37 ± 5.89		
Profession		
Resident	5	31.3
Midwife	10	62.5
Obstetrician-Gynecologist	1	6.3
Years of experience		
0–5	5	31.3
6–15	7	43.8
≥ 16	4	25.0

SD: Standard Deviation

**Table 3:** Themes, sub-themes and quotes of acceptability, adoption, appropriateness, feasibility, and fidelity of birth companions’ implementation strategies

Themes	Sub-themes	Sample quotes
Acceptability	Perceived value of birth companions	“It was a positive experience for me, and I am content with how everything went…”
	Relative advantage	“… I came to this hospital only because I heard that I could have a companion…”
	Credibility	“… The midwife taught me the support techniques… They were effective in relieving my daughter’s pain.”
Adoption	Uptake	“… We offer training to birth companions led by midwives. The midwife teaches…”
	Actual use	“It requires additional time and ongoing monitoring to be effectively integrated into the work tasks of the providers.”
Appropriateness	Perceived usefulness	“I believe it is necessary to have a companion in this maternity hospital due to the overcrowding and insufficient staff…”
	Integration into existing workflows	“… If we use these strategies correctly, there will be no problems in our workflow.”
	Informing women about the possibility of having a birth companion	“It would have been great if they had informed us in advance, so that we could have chosen a more suitable birth companion.”
Feasibility	Training birth companions in prenatal care	“… It is necessary to provide training for labouring women and their companions before they enter maternity hospitals.”
	Recruiting a fixed midwife	“I, the doctor or the midwife, do not have time to train a labouring woman or her companion… The presence of a permanent midwife is important for training.”
	Improving the physical infrastructure	“… It is important to ensure that the physical environment is suitable…”
Fidelity	Adherence	“The providers implemented the program according to the original protocol…”
	Participant responsiveness	“All providers were involved in this program…” “Both women and their companions were receptive to this program…”

### Acceptability

Participants shared opinions on the acceptability of implementing birth companion strategies in three sub-themes: perceived value of birth companions, relative advantage, and credibility.

#### Perceived value of birth companions

Women and birth companions had overall positive experiences with the implementation of birth companions. They believed that the implementation of the program was a good idea, which resulted in continuous support from companions, satisfaction with care, and an improved birth experience. As one woman explained:

*“It was my first delivery, and I was feeling very stressed. The healthcare providers were busy and unable to give me the attention I needed, but having my sister there made a significant difference. She massaged my back, used a hot water bag, assisted me with walking and exercising, and contacted healthcare providers when I required assistance. If my companion was not there, I would have had a difficult birth.”* (Woman 2, 25 years old).

Another person noted that: *“It was a positive experience for me, and I am content with how everything went, particularly because my mother was present in the delivery room. For example, when I was in pain, she would hold my hand and say, ‘send blessings’ or during childbirth, she would say, ‘well done, push, it’s great, I can see the baby’s head’, and it was encouraging … Thank you for making it possible for companions to be with us even during childbirth.”* (Woman 19, 16 years old)

*“The presence of birth companions at this hospital was a good idea; we were satisfied with this program. In the public hospitals of our city, the companions are not allowed to enter the maternity hospital. However, here I had no barrier to my presence …”* (Birth companion 7, 26 years old).

#### Relative advantage

Women were asked if they would be more inclined to choose a hospital for giving birth if it offered birth companions as a standard practice in the maternity ward, and all of them responded affirmatively. One woman stated:

*“When I gave birth a few years ago, they did not allow me to have a companion. This hospital was recommended to me by a friend. She said that last week, my sister gave birth there, and she had a companion… I came here only because I could have a companion, and I was satisfied with having a companion by my side.”* (Woman 14, 21 years old).

#### Credibility

Both women and their companions described the quality of program implementation and training provided by midwives as beneficial:

*“I think this program is being implemented well… The midwife taught me support techniques. I did them for my daughter and tried not to interfere with the clinical work of the providers… They were effective in relieving her pain.”* (Birth companion 14, 50 years old).

*“When I was in pain, my companion used a hot water bag, asked me to take deep breaths, or used Entonox gas… They were very helpful.”* (Woman 12, 24 years old).

While providers also acknowledged the usefulness of implementing birth companions, the implementation team felt that some were initially reluctant to support the program and perceived it as an added burden. However, this reluctance changed over time due to positive outcomes, such as increasing women’s satisfaction, greater participation of companions, and reducing the workload of providers. Several providers also mentioned concerns about limited physical space, violation of women’s privacy, overcrowding, and the transmission of infection:

*“Some of us initially did not support the implementation of this program, because it was perceived as an additional burden. However, after some time of implementation of the program, we observed positive outcomes, such as increased satisfaction among women during childbirth, participation of companions, and a reduction in workload… Now I can confidently say that all the providers have accepted it.”* (Midwife 1, 40 years old).

### Adoption

In this study, adoption of implementation of birth companion strategies was discussed in two sub-themes: uptake and actual use.

#### Uptake

The providers’ responses to the program were positive. They stated that they allow companions to accompany women during labour and childbirth. Upon entering, they provided explanations about the regulations of the maternity hospital, the role and responsibilities of the companion during labour and childbirth. They also taught emotional support techniques such as praying, using calming verbal expressions, encouraging, and comforting. Additionally, they taught physical support techniques including helping with walking, feeding, massaging, and breathing exercises.

*“Upon entering, we ask women if they would like to have a companion. If they wish, we allow their companion to enter the maternity hospital. We teach her (companion)… Finally, we ask her to sign the form to receive training from the midwife.”* (Midwife 14, 29 years old).

*“We allow the companion to be present. We offer training to birth companions led by midwives. The midwife teaches… Most companions also perform well, according to the training they receive.”* (Resident 10, 31 years old).

#### Actual use

Providers’ adoption of the program increased over time as they gained a clearer understanding of how the program was intended to work. However, a few providers also raised concerns that the program may not be sustainable after its initial phase ends. These concerns have contributed to doubts about the program’s full adoption.

*“This program cannot be expected to be sustainable within a few months of implementation… I believe it requires additional time and ongoing monitoring to be effectively integrated into the work tasks of our providers.”* (Head of obstetrics, 49 years old).

### Appropriateness

Participants reported three sub-themes related to the appropriateness of implementation of birth companion strategies, including perceived usefulness, integration into existing workflows, and informing women about the possibility of having a birth companion.

#### Perceived usefulness

Most of the participants agreed that implementation of the program in this maternity hospital was appropriate. One birth companion stated:

*“I believe it is necessary to have a companion in this maternity hospital due to the overcrowding and insufficient staff. The healthcare providers do not have enough time to provide back massages of comfort a woman in labour. As companions, we can fulfil this role for them.”* (Birth companion 4, 46 years old).

#### Integration into existing workflows

Some providers agreed that birth companions could be integrated into the existing workflows:

*“I think that these implementation strategies for birth companions can be very helpful… they are simple and low cost. If we use these strategies correctly, there will be no problems in our workflow.”* (Midwife 3, 41 years old).

#### Informing women about the possibility of having a birth companion

Some women mentioned that if they had been informed in advance (e.g., in childbirth preparation classes) about the possibility of having a birth companion, they could have chosen a more suitable person to accompany them.

*“… If I had known that I could have a companion, I would have brought someone with me who would be more comfortable, trained, or at least had experience with vaginal delivery.”* (Woman 1, 43 years old).

### Feasibility

The providers felt that the routine use of birth companions was feasible in this maternity hospital and described three sub-themes that would contribute to improving feasibility: training birth companions in prenatal care, recruiting a fixed midwife, and improving the physical infrastructure.

#### Training birth companions in prenatal care

The providers commented on the importance of training birth companions and preparing them to play a role in prenatal care. Most providers stated that in order for the few minutes of training upon entering the maternity hospital to be more effective, it is important to give attention to the training of birth companions in childbirth preparation classes.

*“I think the important thing is to train… It is necessary to provide training for labouring women and their companions before they enter maternity hospitals.”* (Midwife 8, 40 years old).

*“… Unfortunately, most of the companions were not trained here. Well, how much time do I have to explain to her during labour?”* (Midwife 13, 37 years old).

*“I believe that training at the maternity hospital can be more effective if the companion is already trained, and our training includes a review component.”* (Resident 11, 30 years old).

#### Recruiting a fixed midwife

Similarly, providers discussed the importance of recruiting a fixed midwife to improve the feasibility of birth companions in maternity hospitals. The majority of providers stated that, in light of the overcrowding and understaffing, successful implementation of the program relied on recruit a fixed midwife who could provide training to labouring women and their companions.

*“… I believe it is necessary to have a permanent midwife for training in order to consistently implement this program.”* (Head of obstetrics, 49 years old).

#### Improving the physical infrastructure

Improving the physical infrastructure of maternity hospitals was also suggested by some providers as a factor related to feasibility:

*“… Yes, routine use of this program is possible, but it is also important to ensure that the physical environment is suitable. We have limited physical space here. The burden of visiting is also high, and we are concerned about overcrowding and the transmission of infection.”* (Resident 2, 30 years old).

### Fidelity

Two sub-themes related to the fidelity of implementation of birth companion strategies were identified: adherence and participant responsiveness.

#### Adherence

Almost all providers agreed that they had implemented the program as intended by the project developers. However, several of them stated that as the implementation progressed, other women (those who were scheduled for a caesarean section or had an abortion) also requested the presence of their companions, which posed a challenge at times. This is because providers had to spend time explaining and justifying their decisions.

*“I believe the providers implemented the program according to the original protocol. I noticed a significant improvement in the conditions at the maternity hospital after the implementation of this program.”* (Midwife 15, 34 years old).

*“… The women who were scheduled for a caesarean section or had an abortion also requested the presence of their companions. If there are also companions, the maternity hospital will become very crowded, which will hinder the provision of proper care.”* (Resident 16, 28 years old).

*“Anyway, when a program starts to reach the ideal, it faces challenges. However, I believe that the providers who were directly involved in the implementation process adhered to the plan…”* (Head of obstetrics, 49 years old).

#### Participant responsiveness

The level of participant engagement in the program was reported to be high, as one provider remarked:

*“I think almost all providers were involved in this program. We may not have had a good participation at the beginning of the program, but it increased over time …”* (Midwife 8, 40 years old).

Furthermore, providers’ statements showed that the reception of women and their companions in the presence of a birth companion was positive:

*“Both women and their companions were receptive to this program. When we informed women that they could have a companion, even during their childbirth, they would be happy…”* (Midwife 4, 41 years old).

## Discussion

This was the first qualitative study in Iran to examine the acceptability, adoption, appropriateness, feasibility, and fidelity of implementation strategies of birth companions based on the experience of women, birth companions, and MHCPs. In summary, the findings of this study indicated strategies for effectively implementing birth companions in public hospitals in Tehran.

In our study, the sub-themes associated with the acceptability of implementing birth companion strategies from the participants’ perspectives included perceived value, relative advantage, and credibility. We found that the implementation strategies used by the birth companion were acceptable to most participants. Our findings are consistent with those of previous studies [33, 34]. Overall, women and their companions greatly appreciated the provision of a birth companion in the hospital, as it improved satisfaction with care and the birth experience [22, 33, 35, 36]. Similarly, providers have described the benefits of implementing birth companions, such as continuous support and a reduced workload [16, 20, 34, 37]. Furthermore, in our study, women and their companions mentioned the benefits of the quality of the program implementation and training provided by midwives. Similar findings have been reported by Kabakian-Khasholian et al. [34].

Our findings showed that although the presence of birth companions was not initially supported by some providers, its acceptance grew over time with an increased understanding of the program as well as the positive outcomes that followed for both women and providers. Another study on birth companions in the labour ward of a center in India showed that providers were initially hesitant to allow birth companions due to overcrowding and the potential disruption of their duties and decision-making [20]. The experience reported by our providers is not surprising. This is an important finding for implementation, and demonstrates that immediate acceptance of new programs after introduction cannot be expected, as research has shown that the acceptability of any program increases with knowledge of that program [25]. A possible explanation for the higher acceptability of birth companions in our study could be attributed to the continuous monitoring of the implementation team and the provision of feedback throughout the implementation process.

The uptake and actual use were perceived as important aspects of adoption of implementing birth companion strategies. Despite the fact that providers adopted the program and responded positively to its use, a few expressed doubts about the program’s sustainability beyond the initial phase. Our findings are consistent with those of a previous study conducted in Arab countries, which reported that obstetric residents expressed uncertainty regarding about the long-term viability of the labour companionship model [34]. Although examining the sustainability of the program was not the goal of our study, it is important to note this issue, which should be explored in the future.

Our study findings showed that the appropriateness of implementation of birth companion strategies refers to the perceived usefulness, integration into existing workflows, and informing women about the possibility of having a birth companion. Providers found that birth companions could be integrated into workflows. Though studies in LIMCs show that providers were reluctant to incorporate birth companions into routine maternity services for reasons such as women’s disobedience to provider instructions, companion interference in care, and the transmission of infections [36, 38, 39]. Some women in our study expressed the desire to be informed about the option of having a birth companion during antenatal care. This finding aligns with a study on birth companionship in Tanzania [33].

This study suggests that the implementation of birth companion strategies in this maternity hospital is feasible, but several potential factors should be considered. Some of our providers pointed out the importance of training birth companions through childbirth preparation classes for the effectiveness of their support upon entering maternity hospitals, as highlighted by Kabakian-Khasholian et al. [34]. Providers also emphasized that recruiting fixed midwives to provide training to women and their birth companions in the maternity hospital was important to support the feasibility of the program. Women and companions have also criticized the infrastructure of the maternity hospital. It is important to note that in this study, any strategy for the reconstruction of physical space (such as the lack of suitable space for the accommodation of companions) was considered but opposed by the management of the maternity hospital, despite it being an important component in the implementation of birth companions.

Our study has several practical implications. Despite the recommendations of the WHO regarding the choice of a companion during labour and childbirth, as well as existing policies, there is a need for the presence of a birth companion in Iran. Increased efforts by policy-makers and managers of maternal health programs are necessary to ensure women’s access to this right and to effectively and sustainably implement it in maternity hospitals. This will help to improve the quality of maternity care and enhance positive childbirth experiences. Furthermore, the collaboration of MHCPs in the implementation of birth companions and the establishment of continuous monitoring systems in maternity hospitals is important. It is also necessary to include training for birth companions in childbirth preparation classes, educating them about their expected role in supporting women.

### Strengths and limitations

To our knowledge, this is the first study to examine the implementation outcomes of birth companions in Iran. This study encompasses a wide range of perspectives and experiences from women, birth companions, and MHCPs. This study has several limitations. First, due to the sensitive nature of the mistreatment issue, participants may have underreported some of their experiences with the companionship program possibly influenced by social desirability bias. We attempted to reduce this bias by conducting interviews in a private room and ensuring the anonymity of the participants’ identities. Second, this study was conducted solely at a public teaching hospital in Tehran, which restricts the generalizability of the findings to private hospitals in Iran. Nonetheless, this study adds to the literature on implementation strategies for birth companion’s support by incorporating implementation research (IR). The findings of this study will be useful for health policymakers in supporting the implementation of birth companions to reduce mistreatment of women during labour and childbirth. However, we recommend continuous monitoring of the actual collaboration among MHCPs during the program implementation process.

## Conclusion

Our study found that the implementation strategies for birth companions in Tehran public hospitals are acceptable, appropriate, and feasible. These strategies improve satisfaction with care and the birth experience, seek continuous support from companions, and reduce provider workloads. However, there are several issues that need to be addressed regarding birth companions in maternity hospitals. These include training birth companions prior to the arrival, informing women about the presence of birth companions, assigning a dedicated midwife to provide training, and improving the physical infrastructure. The findings of this study can be utilized to support the implementation of birth companions in countries with comparable circumstances.

### Electronic supplementary material

Below is the link to the electronic supplementary material.

Supplementary Material 1

Supplementary Material 2

## Data Availability

The datasets generated and analyzed during the current study are not publicly available due to privacy restrictions of the participants but are available from the corresponding author on reasonable request.

## Abbreviations

WHO: World Health Organizaion
RMC: Respectful Maternity Care
EBIs: Evidence-Based Interventions
IS: Implementation Science
MOHME: Ministry of Health and Medical Education
MHCPs: Maternity Healthcare Providers
COREQ: Consolidated Criteria for Reporting Qualitative Research
IR: Implementation Research
