# Supplementary material for: An investigation into the acceptability, adoption, appropriateness, feasibility, and fidelity of implementation strategies for birth companionship in Tehran: a qualitative inquiry on mitigating mistreatment of women during childbirth
Source: BMC Public Health. 2024 May 13;24:1292. doi: 10.1186/s12889-024-18751-z (PMC11089689; doi:10.1186/s12889-024-18751-z)
Supplement: Supplementary file 1 — Supplementary Material 1 [file 12889_2024_18751_MOESM1_ESM.docx]

**Additional file 1: Interview Guides**

1. **Interview Guide for Women**
2. Please provide a description of your overall experience with the implementation of the birth companion program at this hospital.
3. Are you satisfied or dissatisfied with the current implementation of the program or intervention? Why?
4. In your opinion, how appropriate is the implementation of this program or intervention in the hospital?

- What has gone well in its implementation?
- What has gone poorly?

1. In your opinion, can the implementation of this program or intervention help improve women’s childbirth experiences? How? Please explain.
2. If a maternity hospital consistently implemented this program or intervention, would you be more inclined to choose that hospital for giving birth? Why?
3. What changes do you think we should make to make it work better?

Is there anything else you would like to add regarding the topics we discussed today?

1. **Interview Guide for Birth Companions**
2. Please describe your overall experience with the implementation of the birth companion program at this hospital.
3. Are you satisfied or dissatisfied with the implementation of the program or intervention up to this point? Why?
4. In your opinion, how appropriate is the implementation of this program or intervention in the hospital?

- What has gone well in its implementation?
- What has gone poorly?

1. In your opinion, can the implementation of this program or intervention help improve women’s childbirth experiences? How? Please explain.
2. What changes do you think we should make to make it work better?

Is there anything else you would like to add regarding the topics we discussed today?

1. **Interview Guide for Maternity Healthcare Providers and Head of the Maternity Hospital**
2. Please provide an overview of your experience with the implementation of the birth companion program (based on the implementation strategies examined in this study) during the initial phase.
3. Have you used this program or intervention?

- If yes, how did you use it?
- If not, why didn't you use it?

1. How did you experience the use of the program or intervention? Was it positive or negative? Why? Please explain.
2. In your opinion, how appropriate is the implementation of this program or intervention at your hospital?

- What has gone well in its implementation?
- What has gone poorly?

1. In your opinion, was the implementation of this program or intervention at your hospital acceptable? Why? Please explain.
2. Is there anything that we should change to make it more acceptable and appealing to your hospital?
3. Did you encounter any issues in your workflow during implementation? Please explain.
4. In your opinion, can the implementation of this program or intervention be beneficial for improving the quality of care and enhancing women's experiences of childbirth? Please explain.
5. How, in your opinion, has the reception of pregnant women and their companions been? Were they receptive to the program or intervention? Why?
6. What are your thoughts on integrating this program or intervention into your hospital?

- What makes it difficult to implement?
- What facilitates the implementation of it?

1. Did maternity healthcare providers implement the program or intervention as intended by project developers?

- If not, why?
- What changes should be made in this regard?

Is there anything else you would like to add regarding the topics we have discussed today?
